# Ligands of Biological and Environmental Interest as Sequestering Agents for Fe^3+^ in Aqueous Solution: A Speciation Study of Natural Fluids

**DOI:** 10.3390/molecules30142991

**Published:** 2025-07-16

**Authors:** Anna Irto, Ileana Ielo, Clemente Bretti, Francesco Crea, Concetta De Stefano, Rosalia Maria Cigala

**Affiliations:** Dipartimento di Scienze Chimiche, Biologiche, Farmaceutiche ed Ambientali, Università di Messina, Viale F. Stagno d’Alcontres, 31-98166 Messina, Italy; airto@unime.it (A.I.); ilielo@unime.it (I.I.); cbretti@unime.it (C.B.); cdestefano@unime.it (C.D.S.); rmcigala@unime.it (R.M.C.)

**Keywords:** Iron(III), Tranexamic acid, Indole-3-acetic acid, Aminomethylphosphonic acid, Sequestration

## Abstract

The interactions of Fe^3+^ with some ligands (Tranexamic (*TXA^−^*), Indole-3-acetic (*IAA^−^*), and Aminomethylphosphonic (*AMPA*^2−^) acids) of biological and environmental interest were studied. The speciation studies were performed in NaNO_3(aq)_ and NaCl_(aq)_ using potentiometric and, only for *IAA*^−^, spectrophotometric titrations at *T* = 298.15 K and 0.01 ≤ *I*/mol dm^−3^ ≤ 1.0. The proposed speciation models are as follows: Fe(*TXA*)H^3+^, Fe(*TXA*)_2_^+^, Fe(*TXA*)(OH)^+^, and Fe(*TXA*)(OH)_2(aq)_ for *TXA*^−^; Fe(*IAA*)^2+^ for *IAA*^−^; and Fe(*AMPA*)H_2_^3+^, Fe(*AMPA*)H^2+^, and Fe(*AMPA*)^+^ for *AMPA*^2−^. A comparison of log*β* for the common Fe*L* species gives log*β_FeIAA_* = 6.56 and log*β_FeAMPA_* = 14.84 (at *I* = 1.00 mol dm^−3^ and *T* = 298.15 K), suggesting that *AMPA*^2−^ has a higher complexing ability towards Fe^3+^ than *IAA*^−^. The dependence on the ionic strength of the formation constants was modeled by means of a Debye–Hückel type equation and the SIT model, whilst the sequestering ability of the investigated ligands towards Fe^3+^ was quantified at various pHs, ionic strengths, and in the different supporting electrolytes by means of an empirical pL_0.5_ parameter. To complete this study of the behavior of the different Fe^3+^/ligand systems, various simulations in biological fluids and natural waters were conducted.

## 1. Introduction

In natural systems, metal cations tend to interact with active molecules of an organic nature, forming complexes of diverse stoichiometry and various chemical–physical properties. The different stoichiometry of the metal–ligand species can influence processes such as transport, absorption, and excretion processes, as well as the distribution between the various organs [1,2,3,4].

Since natural systems are multicomponent solutions containing numerous organic and inorganic components at different concentrations, and their interactions with metal can lead to the formation of complex species, an alteration of their chemical–physical properties can be observed.

During the formulation and delivery of a drug, knowledge of the thermodynamic properties of metals and ligands (i.e., hydrolysis, protonation, and complexation constants) is a fundamental factor in understanding how a pharmaceutical substance can act in body fluids and/or natural waters, allowing for the development of new analytical methods and technological processes to improve the absorption and/or remove these components. Studies performed in blood plasma or in aqueous solutions that simulate biological fluids are a useful tool for understanding the in vivo biochemical behavior of both essential and toxic metals. Furthermore, they can offer a fundamental basis for the evaluation of the effectiveness of classes of chelators in removing metals from the body.

The presence of biologically active molecules in natural systems is not only of biological relevance, but also of environmental concern; in fact, it is well known that many drugs are currently present in high quantities in natural waters, making them emerging contaminants. Therefore, there is currently a need to find solutions for the removal of these molecules from the natural environment.

**Scheme 1:**
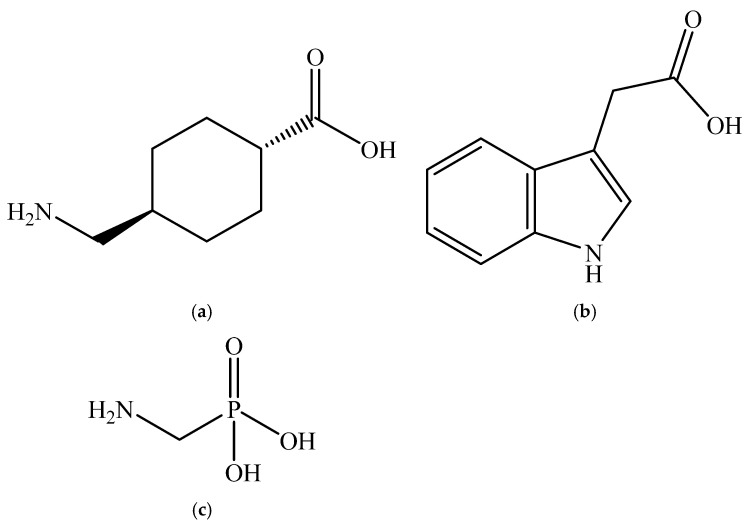
Structure of tranexamic acid (*TXA^−^*) (**a**), indole-3-acetic acid (*IAA^−^*) (**b**)**,** and aminomethylphosphonic acid (*AMPA*^2−^) (**c**).

In this paper, the results of speciation studies of molecules of biological, clinical, and environmental interest, such as tranexamic acid (*TXA^−^*), indole-3-acetic acid (*IAA^−^*), and aminomethylphosphonic acid (*AMPA*^2*−*^), are reported (Scheme 1).

Tranexamic acid is a synthetic derivative of the amino acid lysine, employed in the formulation of antifibrinolytic drugs that control bleeding, help blood to clot, and are often used for nosebleeds [5,6,7].

Indole-3-acetic acid is a protein-bound indolic uremic toxin and an index of disease as enhanced tissue factor synthesis in endothelial and peripheral blood mononuclear cells, endothelial inflammation, and oxidative stress lead to a higher risk of thrombotic events and both cardiovascular and all-cause mortality. *IAA^−^* is produced by intestinal bacteria upon metabolizing tryptophan. Furthermore, this acid has an important impact on the environment, as reported in [8,9,10,11].

Aminomethylphosphonic acid is the main metabolite of the widely used pesticide, glyphosate; its toxicity is similar to the mentioned precursor, and is therefore of considerable analogous toxicological concern [12,13,14].

Particular attention is paid to the interaction of these three ligands with Fe^3+^; investigations were performed in an aqueous solution containing different supporting electrolytes at various ionic strengths and temperatures by means of potentiometry and UV-Vis spectrophotometry (only for *IAA^−^*) at *T* = 298.15 K.

The high stability of the Fe^3+^/*AMPA*^2−^ complexes was confirmed by means of exchange measurements, employing a competitive ligand, namely EDTA [15,16,17]; the results obtained from these two different approaches can be considered in perfect agreement in terms of the stability of the complexes.

The data presented in this paper are of fundamental importance for both biological and environmental problems due to the overloading of Fe^3+^ and because knowledge of the thermodynamic parameters of these interactions can be useful in optimizing the transport or remediation procedures of these components.

## 2. Results and Discussion

### 2.1. Hydrolytic Behavior of Iron(III)

The hydrolytic constants of Fe^3+^ in NaCl_(aq)_ and NaNO_3(aq)_ at the desired experimental conditions were taken from a previous paper [18].

### 2.2. Tranexamic Acid

The acid–base properties of tranexamic acid (*TXA^−^*) can be attributed to the carboxylic group on the cyclohexane ring and to the amino one on the methylene in *para* to the carboxylic group. The tranexamic protonation constants were already studied by this research group at different ionic strengths and in various ionic media (unpublished data from this laboratory). As for the hydrolysis constant values of Fe^3+^, the protonation data of the ligand were also used to define a correct speciation model for the interaction between Fe^3+^ and *TXA^−^*. Since a known amount of standard HCl solution was added to the measurement vessel in order to adjust the initial pH value at ~ 2, the weak FeCl^2+^, FeCl_2_^+^, and FeCl(OH)^+^ complexes [18,19] were also considered in the speciation model.

The Fe^3+^/*TXA*^−^ interactions were investigated in NaNO_3_ aqueous solutions at 0.15 ≤ *I*/mol dm^−3^ ≤ 1.00 and *T* = 298.15 K using the potentiometric technique. The experimental details are already reported in Table S1 of the Supporting Information. The investigated pH range was limited by the formation of a sparingly soluble species; the pH of formation of the insoluble species depended both on the Fe^3+^ concentration and on the metal–ligand molar ratios, and never exceeded pH 5.3.

The best results were obtained considering the following speciation model: Fe(*TXA*)H^3+^, Fe(*TXA*)_2_^+^, Fe(*TXA*)(OH)^+^, and Fe(*TXA*)(OH)_2(aq)_. The formation constants of the Fe^3+^/*TXA^−^* complexes, determined by means of the BSTAC computer program [20] and expressed by the general Equations (2) and (3), are reported at the different ionic strengths in Table 1.

Figure S1 reports a distribution diagram of the Fe^3+^/*TXA^−^* species at *I* = 0.15 mol dm^−3^ (NaNO_3(aq)_) and *T* = 298.15 K. The free metal cation percentage tends to decrease with the formation of the Fe(*TXA*)H^3+^ species, which reaches approximately 40% at pH~2.5, while the Fe(OH)^2+^ hydrolytic species achieves 12%. The Fe(*TXA*)OH^+^ and Fe(*TXA*)_2_^+^ complexes reach 22 and 18% of formation at pH~3.4 and 4.4, respectively. The Fe(*TXA*)(OH)_2(aq)_ is the most important species, since it achieves 80% at pH~5.3. Even if the formation of the Fe^3+^/*TXA*^−^ species tends to avoid the hydrolysis of the metal cation, the Fe_12_(OH)_34_^2−^ reaches ~50% at pH~3.5.

The weak FeCl^2+^, FeCl_2_^+^, and FeCl(OH)^+^ complexes are present at pH~2.3, but with percentages that never exceeded 5% due to the low Cl^−^ concentration in the vessel.

### 2.3. Indol-3-Acetic Acid

Owing to the formation of precipitates at the experimental conditions (i.e., concentration of the components) of potentiometric titrations, the Fe^3+^/*IAA*^−^ system was studied using spectrophotometry.

The Fe^3+^/indol-3-acetic acid titrations were performed at different components concentrations in absence of ionic medium (*I* = 0.01 mol dm^−3^ in HCl_(aq)_) and at 0.15 ≤ *I*/mol dm^−3^ ≤ 1.00 in NaCl_(aq),_ *T* = 298.15 K.

The analysis of the experimental absorption spectra, reported as examples in Figure S2a,b at two different ionic strengths, showed the formation of an absorption band with λ_max_ = 277 nm at pH~2.10 that increased up to the final investigated pH.

The elaboration of the experimental data was performed using the HypSpec computer program [21] in the wavelength range 210 ≤ λ/nm ≤ 500, taking into account the ligand protonation (3-indoleacetic acid = *L*^−^) as well as its UV-Vis behavior (unpublished data from this laboratory) and the metal cation hydrolytic constants [18]. The data analysis was performed considering the entire pH range investigated, but the best results in terms of the speciation model and statistical parameters were obtained considering the pH range 2.0–7.0 and only for the Fe(*IAA*)^2+^ species. The formation of Fe(*IAA*)_2_^+^ and hydrolytic mixed Fe(*IAA*)OH^+^ complexes was also checked, but they were rejected by the HypSpec program or their formation percentages never exceeded 1–2%. For this reason, these species were neglected. The formation constants determined at the different ionic strengths are reported in Table 1. The distribution diagram represented in Figure S3 at *I* = 0.15 mol dm^−3^ in NaCl_(aq)_ shows that the Fe(*IAA*)^2+^ species reaches ~30% at pH~2.8, while at pH ≥ 4.5, only the metal hydrolytic species are present.

The literature reports only the information published by Recaldin and Heath [22] on the Fe^3+^/*IAA*^−^ system; they conducted spectrophotometric investigations at *c*_Fe_ = 4.0 mmol dm^−3^ and *c*_IAA_ = 2.0, 4.0, 100.0 mmol dm^−3^, in a solvent mixture consisting of 50% ethanol and 50% water and in absence of an ionic medium. The authors determined, for the Fe(*IAA*)^2+^ species, by means of the Bjerrum method, a formation constant value of log*K*_110_ = 6.0, quite similar to the result reported in Table 1, despite the different experimental conditions of the two investigations.

### 2.4. Aminomethylphosphonic Acid

The acid–base properties of the aminomethylphosphonic acid (*AMPA*^2−^), the main metabolite of the glyphosate, are related to the protonated amino group (–NH_2_ to –NH_3_^+^) and the phosphonate one [23,24]. The acid–base properties of the ligand were studied in NaCl_(aq)_ at different ionic strengths and *T* = 298.15 K. Here, the complexation of Fe^3+^ by *AMPA*^2−^ were studied using potentiometry in NaCl_(aq)_ at 0.10 ≤ *I*/mol dm^−3^ ≤ 1.0 and *T* = 298.15 K; the pH range of investigation was limited by the formation of a sparingly soluble species at pH~5.3, as reported by Barja et al. [25]. For more details on the experimental conditions, see Table S1.

The proposed speciation scheme is featured by the Fe(*AMPA*)H_2_^3+^, Fe(*AMPA*)H^2+^, and Fe(*AMPA*)^+^ species, and the corresponding overall formation constants at different ionic strengths expressed by means of the equilibria (2) and (3) are reported in Table 1.

Figure S4 shows the distribution diagram of the Fe^3+^/*AMPA*^2−^ species in NaCl_(aq)_ and *I* = 0.10 mol dm^−3^. The complexes formation hampers the hydrolysis of the metal cation; the Fe(*AMPA*)H_2_^3+^ and Fe(*AMPA*)H^2+^ species represent the predominant complex, reaching ~93% of formation at pH~2.0 and 4.6, respectively, while the Fe(*AMPA*)^+^~9.0% at pH~5.0.

Due to the apparently high stability of Fe^3+^/*AMPA*^2−^ complex species (as an example, at *I* = 0.105 mol dm^−3^ log*K*_Fe(*AMPA*)+_ = 15.07) and the almost total complexation of the metal at the begin of the titration, to validate the obtained results, an alternative approach, often employed in similar cases, was used. To apply this second approach, it is necessary to use a second competitive ligand (here, Ethylenediaminetetraacetic acid, *EDTA*, was used), able to interact with the metal (Fe^3+^) forming metal–ligand species of similar stability to those formed with the first ligand, in order to avoid the 100% of metal complexation at low pH values. The first ligand (A*MPA*^2−^) has to be removed using a displacement reaction, *EDTA*. Further details on the use of the competitive ligands are reported in the literature [15,16,17].

Obviously, both the acid–base properties of *EDTA* and the interactions with Fe^3+^ at the desired experimental conditions must be known. The literature reports that the main complex species of the Fe^3+^/*EDTA*^4−^ system are the Fe(*EDTA*)H_(aq)_ and Fe(*EDTA*)^−^, with log*β* of 26.1 and 24.8 [26], at *I* = 0.1 in KCl_(aq)_ and *T* = 298.15 K, respectively.

Competitive experiments were performed at *I* = 0.15 mol dm^−3^ (NaCl_(aq)_), *T* = 298.15 K, and 1.0 ≤ *c*_Fe_/mmol dm^−3^ ≤ 2.0, 1.0 ≤ *c*_AMPA_/mmol dm^−3^ ≤ 4.1, whilst for *EDTA* 1.0 ≤ *c*_EDTA_/mmol dm^−3^ ≤ 4.0. Different molar concentration ratios in favor, mainly, of *AMPA* were considered. The experimental data was processed, also taking into account the acid–base properties of the metal, *AMPA* and *EDTA*, as well as their complexation towards Fe^3+^. The speciation model obtained by using this second approach is equal to the one previously proposed, Fe(*AMPA*)H_2_^3+^, Fe(*AMPA*)H^2+^, and Fe(*AMPA*)^+^ species, as well as the stability of the complexes (log*β* = 23.8 ± 0.3, 19.6 ± 0.4, and 14.8 ± 0.3, respectively). The differences in the values can be justified considering the various experimental conditions used for the two approaches (*I* = 0.15 mol dm^−3^ for the simple system and *I* = 0.10 mol dm^−3^ for the competitive measurements).

### 2.5. Stability Constants at Infinite Dilution and Parameters for the Dependence on Ionic Strength

The dependence on the ionic strength of the formation constants of the Fe^3+^/ligand species was modeled by means of the Debye–Hückel-type equation (Equation (4)) and the SIT approach (Equation (6)) reported in the Section 3.4.

The thermodynamic formation constants at infinite dilution, the calculated values at different ionic strengths, and the *C* and Δ*ε* parameters for the dependence on *I*/mol dm^−3^ in NaNO_3(aq)_ and NaCl_(aq)_, *T* = 298.15 K, are listed in Table 2.

From the data reported in Table 2, it is difficult to make a comparison between the stability of the species formed by Fe^3+^ with the ligands, since different speciation models were proposed. Analyzing the common Fe*L* species obtained for the two systems studied in NaCl_(aq)_, it is possible to observe that, since the complexes are formed by electrostatic interactions, their stability depends on the charges involved in the formation reactions. The trend is log*K*_FeL_: *AMPA*^2−^ >> *IAA*^−^. These differences can be explained comparing the z* parameter (see Equation (5)), whose value depends on the charges of the reagents and products of reaction, as well as on the stoichiometric coefficients involved in the reaction of formation of a given species; as an example, considering the formation of the Fe*L* species, z* is 6 for *IAA*^−^ and 12 for *AMPA*^2−^, respectively.

The conversion of the stability constants and ionic strength from the molar to the molal concentration scale allowed for the application of the SIT approach to model the variation in the log*β* values vs. *I_m_*/mol kg^−1^(H_2_O) and to calculate the specific ion interaction coefficients, as reported in Table 2.

### 2.6. Sequestering Ability of the Ligands Towards Fe^3+^

In numerous previous articles, a Boltzmann-type equation was widely employed to quantify the sequestering capacity of one or more ligands towards a metal cation [27]. This ability can be expressed by means of the pL_0.5_ parameter [27] reported in Equation (1), calculated using a dose–response type equation, which takes into account the sum of the molar fractions (*χ*_M_) of all the metal–ligand complexes species vs. the co-logarithm of the total ligand concentration (pL).

(1) $$\chi_{M}=\frac{1}{1+10^{(\mathrm{pL}-{\mathrm{pL}}_{0.5})}}$$

This function can be graphically represented as a sigmoidal curve with asymptotes of 1 for p*L* → −∞ and 0 for p*L* → +∞.

The sequestering ability of *TXA*^−^, *IAA*^−^, and *AMPA*^2−^ towards Fe^3+^ was evaluated by calculating the pL_0.5_ at different pH and ionic strength values in NaCl_(aq)_ and NaNO_3(aq)_, as reported in Table S2 of the Supporting Information.

The different structures (Scheme 1) and acid–base properties of the ligands seem to influence the Fe^3+^ sequestration. At each ionic strength value, the sequestering ability of *TXA*^−^ towards Fe^3+^ tends to increase with the pH.

Concerning the 3-indoleacetic acid, at *I* = 0.15 mol dm^−3^, the sequestration raises with pH up to pH~3.0, then it decreases, and, from pH~6.0, it assumes negligible values, possibly owing to the presence of only metal hydrolytic species (Fe(OH)_2_ and Fe_12_(OH)_34_). At other ionic strengths, the pL_0.5_ values are insignificant at pH ≥ 6.0.

For the Fe^3+^/*AMPA*^2−^, a lowering of the pL_0.5_ is observed, increasing the ionic strength. This behavior could be explained by the possible formation of the FeCl_i_^(3−i)^ species and the competition between Fe^3+^ and Na^+^ (especially at high ionic strengths) to interact with the ligand. Considering a common experimental condition for all the investigated systems, namely pH = 5.0, *I* = 1.00 mol dm^−3^, and *T* = 298.15 K, the pL_0.5_ values reported in Table S2 shows the following trend: *AMPA*^2−^ (4.89) > *TXA*^−^ (4.00) > *IAA*^−^ (2.15), also observable in Figure S5.

### 2.7. Simulations in Natural Waters and Biological Fluids

The knowledge of the thermodynamic formation parameters has a fundamental role in problems related to different fields, such as the industrial, biological (metal detoxification or drug delivery), and environmental fields (remediation of contaminated sites). Moreover, it gives the possibility of knowing not only the species in which a given component is distributed in a real system (biological or natural), but also their abundance at certain concentrations of the components, pHs, temperatures, etc., information that cannot be obtained from the sole knowledge of its analytical concentration (i.e., total). Furthermore, the knowledge of thermodynamic parameters and accurate speciation study also allows us to consider the “weight” that secondary components and reactions may have on the formation of the species of the studied system and on their distribution.

Considering the importance that the ligands here studied may have in both biological and environmental fields, it was considered useful to carry out simulations that would allow us to understand how the species of the Fe^3+^/ligand systems are distributed when present in conditions simulating those of real systems, such as saliva, urine, or plasma for biological systems, and acid rainwater and seawater for the environmental systems.

In these cases, the input models used to simulate the behavior of the Fe^3+^/ligand systems also consider the main components (organic and inorganic) naturally present and their average concentrations. The formation constants of all the secondary species that these components can form were also considered (for further details, see Tables S3–S6 of the Supporting Information).

Some distribution diagrams were drawn to simulate the behavior of the Fe^3+^/Ligand systems.

**Figure 1:**
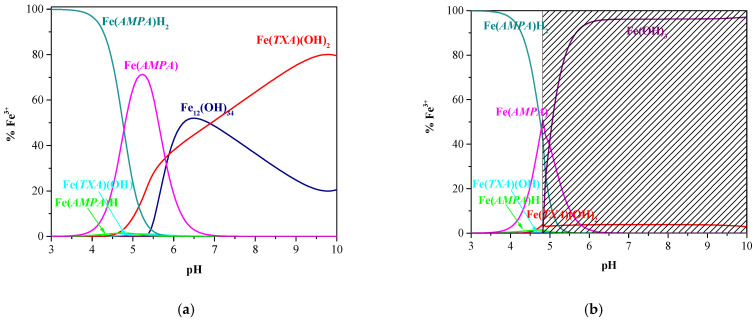
Distribution diagram of the Fe^3+^/ligand species at *I* = 0.10 mol dm^−3^ in NaCl_(aq)_ and *T* = 298.15 K (**a**) without considering the Fe(OH)_3(s)_ formation; (**b**) considering the Fe(OH)_3(s)_ formation. Experimental conditions: *c*_Fe_ = 1.5 mmol dm^−3^; *c_TXA_* = 2.2 mmol dm^−3^; *c_IAA_* = 2.2 mmol dm^−3^; *c_AMPA_* = 4.0 mmol dm^−3^ (charges omitted for simplicity).

Figure 1a,b reports two different graphs. The first one is a distribution diagram drawn considering contemporary all the ligands in the same system and neglecting the possible formation of the Fe(OH)_3(s)_ species. The different Fe^3+^/ligand species are distributed in the whole pH range, with a prevalence of the Fe^3+^/*AMPA*^2−^ and Fe^3+^/*TXA*^−^ ones. The Fe^3+^/*AMPA*^2−^ species, in particular the Fe(*AMPA*)H_2_^+^ and Fe(*AMPA*)^+^, are observable only at acidic pHs, while the Fe(*AMPA*)H^2+^ is present in negligible quantities. For the Fe^3+^/*TXA*^−^ complexes, only the Fe(*TXA*)(OH)_2_^0^_(aq)_, is present in almost all pH ranges investigated. Concerning this diagram, some considerations must be acknowledged; the first one is the absence (at the simulated conditions) of the Fe^3+^/*IAA*^−^ species due to the low stability of its species with respect to the other systems, and, in particular, the formation of the Fe(*AMPA*)H_2_^+^ species at acidic pHs, prevents the formation of the Fe(*IAA*)^2+^ complex.

A second simulation was performed including in the speciation scheme the solubility product of Fe(OH)_3(s)_ (Figure 1b), whose solubility product [19] was considered in the input. The distribution of the species changes at pH~4.8, where the species simulation indicates the formation of the insoluble species and the Fe(*TXA*)(OH)_2_^0^_(aq)_ disappears.

**Figure 2:**
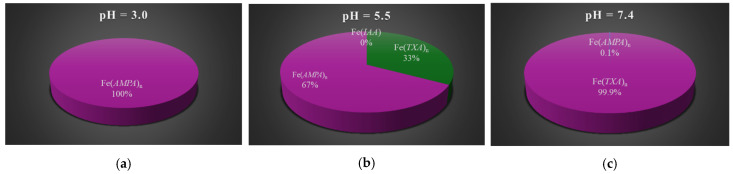
(**a**–**c**). Pie charts at different pH values with the sum of the species of the Fe^3+^/ligand systems at *I* = 0.10 mol dm^−3^ in NaCl_(aq)_ and *T* = 298.15 K. Component concentrations: *c*_Fe_ = 1.5 mmol dm^−3^; *c_TXA_* = 2.2 mmol dm^−3^; *c_IAA_* = 4.0 mmol dm^−3^; *c_AMPA_* = 2.2 mmol dm^−3^.

Obviously, these considerations are valid at the simulated conditions, since varying the components concentrations, the distribution and percentage of formation of the species vary. However, these simulations can be useful to highlight at the different pHs of different fluids and the abundance of the different species, as reported in Figure 2a–c, where different pie charts, obtained considering the sum of the species of each Fe^3+^/ligand system at *I* = 0.10 mol dm^−3^ in NaCl_(aq)_ and *T* = 298.15 K, are presented.

Figure 2a can simulate an industrial wastewater at pH = 3.0; in those conditions, Fe^3+^ is complexed by *AMPA*^2−^ (100%). At pH = 5.5 (possible pH of rainwater), the Fe^3+^/*AMPA*^2−^ species decrease (67%) and the Fe^3+^/*TXA*^−^ species become significant (33%); at pH = 7.4 (blood plasma), the Fe^3+^/*TXA*^−^ species are predominant, and for the Fe^3+^/*AMPA*^2−^ species, only 0.1% of formation was obtained. In all conditions, the formation percentage of the Fe^3+^/*IAA*^−^ is negligible.

**Figure 3:**
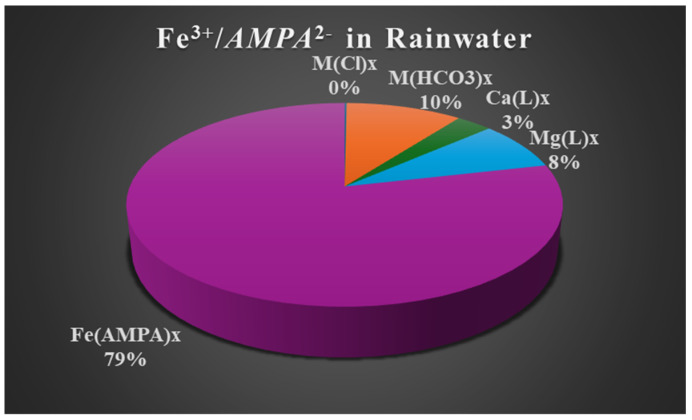
Pie chart with the sum of the Fe^3+^/*AMPA*^2−^ species and of those formed by the secondary components at *I* = 0.01 mol dm^−3^ in NaCl_(aq)_, pH = 5.5, and *T* = 298.15 K. Component concentrations: *c*_Ca_ = 0.02 mmol dm^−3^; *c*_Mg_ = 0.05 mmol dm^−3^; *c*_Cl_ = 0.48 mmol dm^−3^; *c*_Na_ = 0.41 mmol dm^−3^; *c*_HCO3_ = 0.065 mmol dm^−3^; *c*_Fe_ = 0.5 mmol dm^−3^; and *c_AMPA_* = 1.0 mmol dm^−3^.

A pie chart graph can be obtained simulating the condition of real rainwater [28]; in this case (see Figure 3), the input model is more complex, since it contains, other than the hydrolytic constants of Fe^3+^ and the protonation constants of the ligand (*AMPA*^2−^, in this case), the main components of the rainwater, their concentrations (*c*_Ca_ = 0.02 mmol dm^−3^; *c*_Mg_ = 0.05 mmol dm^−3^; *c*_Cl_ = 0.48 mmol dm^−3^; *c*_Na_ = 0.41 mmol dm^−3^; and *c*_HCO3_ = 0.065 mmol dm^−3^), and the stability constants of the species formed by their internal interactions. For the simulation in Figure 3, the following concentrations of Fe^3+^ and *AMPA*^2−^ were used: *c*_Fe_ = 0.5 mmol dm^−3^ and *c_AMPA_* = 1.0 mmol dm^−3^. Table S3 of the Supporting Information reports the stability constants of the equilibria involving the main natural components of acidic rainwater at *I* = 0.010 mol dm^−3^ (estimated mean ionic strength value of rainwater) and *T* = 298.15 K.

As it can be seen from Figure 3, at pH = 5.5, the Fe^3+^/*AMPA*^2−^ species are the main ones, whilst those formed by *AMPA*^2−^ with Ca^2+^ and Mg^2+^ reach 3% and 8%, respectively. The sum of all the bicarbonate species (with Na^+^, Ca^2+^, Mg^2+^) achieves ~ 10%, while those formed by Cl^−^ are negligible.

Concerning the simulation in urine, the system is much more complicated, owing to the higher number of components and equilibria of formation to be considered. Human urine is composed primarily of water (95%). The rest is made up of urea (2%), creatinine (0.1%), uric acid (0.03%), chloride, sodium, potassium, sulfate, ammonium, phosphate, and other ions and molecules in lesser amounts. For the simulation of urine, a model proposed by Sarigul et al. [29] was used; for more details on the stability constants of the species, see Table S4.

**Figure 4:**
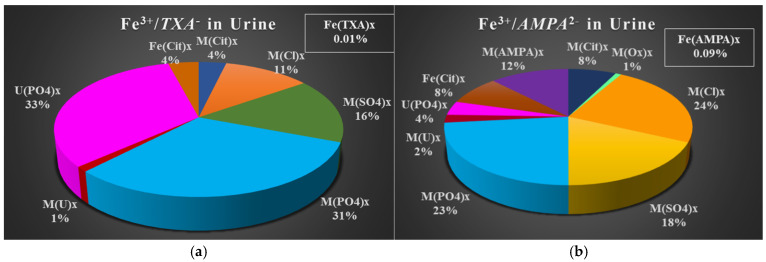
(**a**,**b**). Pie charts with the sum of the Fe^3+^/ligand species and of those formed by the secondary components in urine [29] at pH = 5.0. Components concentrations: *c*_Na_ = 54.4 mmol dm^−3^; *c*_K_ = 31.03 mmol dm^−3^; *c*_Ca_ = 1.66 mmol dm^−3^; *c*_Mg_ = 4.39 mmol dm^−3^; *c*_NH4_ = 2.37 mmol dm^−3^; *c*_Cl_ = 85.7 mmol dm^−3^; *c*_SO4_ = 16.35 mmol dm^−3^; *c*_PO4_ = 23.33 mmol dm^−3^; *c_Uric acid_* = 1.49 mmol dm^−3^; *c_Urea_* = 250 mmol dm^−3^; *c*_Ox_ = 0.19 mmol dm^−3^; *c_Cit_* = 2.45 mmol dm^−3^; *c*_Fe_ = 1.0 mmol dm^−3^; and *c_Ligand_* = 2.0 mmol dm^−3^.

Some simulations in urine were made for each Fe^3+^/ligand system, as reported in Figure 4a,b.

Taking into account the urine model proposed by Sarigul et al. [29], a system featuring six cations and seven anions was obtained. From the internal interactions between the components of urine, 94 different equilibria must be considered. Owing to the complexity of the system and the concentration of the components, both binary and ternary internal interactions between the urine components (as an example, NaK*Cit*H; NaNH_4_(*Cit*)H; KNH_4_(PO_4_)H, etc.) must be considered [30], as well as the interactions of (Ca^2+^, Mg^2+^, Na^+^, K^+^) with *AMPA*^2−^ and *TXA*^−^. Similarly, for Fe^3+^, the interactions with citrate, urea, uric acid, phosphate, sulfate, and chloride must be taken into account. In total, the speciation scheme is formed of 125 different complexes (including the protonation of the ligand, the hydrolysis of the metal, and their interactions).

Analyzing the pie charts reported in Figure 4a,b, it is possible to observe that, for all the systems, the percentage of Fe^3+^/ligand complexes species can be considered negligible (<5%).

Similar simulations can be performed in other biological fluids such as saliva and human blood plasma. In the first case, as already seen for urine, the number of species and equilibria to be considered is high. The literature reports a simplified approach to study the acid–base and complexing properties of saliva [30], where this multicomponent solution has been considered as a MX single salt with mean charge of components equal to ±1.163, where M is representative of all the main dissolved cations of saliva and X of the anions. By using this approach, only 4 equilibria, instead of 93, are sufficient to represent the acid–base and complexing properties of the saliva components. The components to be used for the preparation of synthetic saliva and their corresponding concentration were taken from [30,31].

However, for the present investigations, all the internal concentrations of each component were considered. Table S5 of the Supporting Information reports all the equilibria considered in the input of the HySS program and those obtained from [30], in addition to the Fe^3+^/ligand, the Fe^3+^ hydrolysis, and protonation of the ligands.

**Figure 5:**
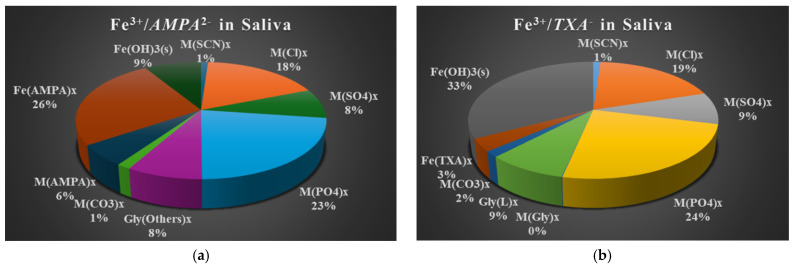
(**a**,**b**). Pie charts with the sum of the Fe^3+^/ligand species and of those formed by the secondary components in saliva [30] at pH = 5.0. Component concentrations: *c*_Na_ = 20.0 mmol dm^−3^; *c*_K_ = 28.9 mmol dm^−3^; *c*_Ca_ = 2.1 mmol dm^−3^; *c*_Mg_ = 0.5 mmol dm^−3^; *c*_NH4_ = 3.5 mmol dm^−3^; *c*_Cl_ = 25.3 mmol dm^−3^; *c*_SO4_ = 1.1 mmol dm^−3^; *c*_PO4_ = 8.5 mmol dm^−3^; *c_Urea_* = 3.33 mmol dm^−3^; *c*_CO3_ = 11.4 mmol dm^−3^; *c*_SCN_ = 1.95 mmol dm^−3^; *c_Gly_* = 0.35 mmol dm^−3^; *c*_F_ = 0.0025 mmol dm^−3^; *c*_Fe_ = 1.0 mmol dm^−3^; and *c_Ligand_* = 2.0 mmol dm^−3^.

Analyzing the pie charts in Figure 5a,b, obtained in saliva at pH = 5.0, some considerations can be acknowledged; independent of the Fe^3+^/ligand system, the formation percentage of the species formed by the interaction of Fe^3+^ with the main inorganic components of saliva (i.e., phosphate, chloride, sulfate, and carbonate) are very similar, with a prevalence of the phosphate complexes that reach ~24% and the chloride ones (~18–19%). Very low, ~1%, are the sum of the formation percentages of the carbonate and thiocyanate species.

Concerning the ligands here investigated, in addition to the species with Fe^3+^, in the pie charts, there is the presence of the M/Ligand species (M = Ca^2+^, Mg^2+^, Na^+^, *etc*), except for *TXA*^−^; in the case of Fe^3+^/*AMPA*^2−^ system, the formation percentages do not exceed 6%. The Fe^3+^/ligand complexes follow the trend Fe(*AMPA*)_x_ 26% >> Fe(*TXA*)_x_ 3% (with Fe(*IAA*)_x_ = 0 in all cases).

Moreover, by observing the pie charts of Figure 5a,b, another important piece of information can be obtained, namely, that at the condition of the simulation *c*_Fe_ = 1.0 mmol dm^−3^ and *c_ligand_* = 2.0 mmol dm^−3^, the formation of the Fe(OH)_3(s)_ insoluble species was observed, reaching, for the *AMPA*^2−^ system, 9% of formation and 33% for the *TXA*^−^ one.

In the case of Fe^3+^/*AMPA*^2−^ system, at Fe^3+^ concentrations lower than *c*_Fe_ = 0.3 mmol dm^−3^, the formation of the insoluble species disappears, whilst for Fe^3+^/*TXA*^−^, it is necessary a Fe^3+^ concentration < 0.1 mmol dm^−3^.

For simulations in human blood plasma, the literature reports different models, in which several organic and inorganic components are considered. For the simulation here performed, the model proposed by Marques et al. [32] was employed. This model considers the presence of various inorganic salts (sodium chloride, sodium bicarbonate, potassium chloride, dibasic potassium phosphate trihydrate, magnesium chloride hexahydrate, calcium chloride dihydrate, sodium sulfate), while the organic fraction is simulated by the presence of tris(hydroxymethyl)aminomethane (*THAM*).

Taking into account all the possible interactions (binary and ternary) between the internal components of plasma, 58 different equilibria and their corresponding formation constants (for the ligands protonation and complexes formation) must be considered; the hydrolysis of the metal cations (i.e., Na^+^, K^+^, Ca^2+^ and Mg^2+^) were neglected since the reactions occur at pH > 10. More details are reported in Table S6 of the Supporting Information.

**Figure 6:**
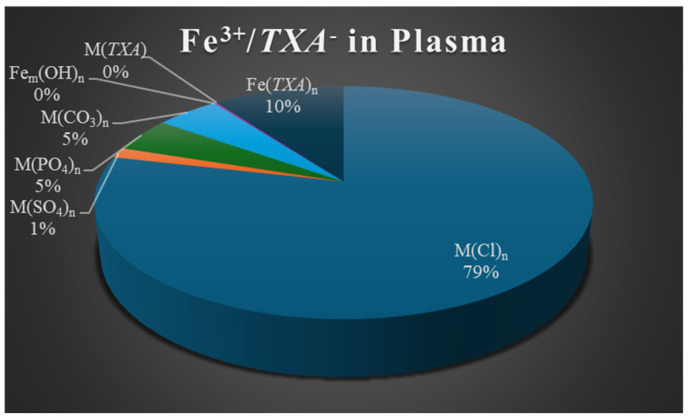
Pie chart with the sum of the Fe^3+^/*TXA*^−^ species and of those formed by the secondary components in human plasma [32] at pH = 7.4. Component concentrations: *c*_Na_ = 115.9 mmol dm^−3^; *c*_K_ = 4.2 mmol dm^−3^; *c*_Ca_ = 1.6 mmol dm^−3^; *c*_Mg_ = 1.2 mmol dm^−3^; *c*_Cl-_ = 170.2 mmol dm^−3^; *c*_SO4_ = 0.4 mmol dm^−3^; *c*_PO4_ = 0.9 mmol dm^−3^; *c*_CO3_ = 3.4 mmol dm^−3^; *c_THAM_* = 31.4 mmol dm^−3^; *c*_Fe_ = 1.0 mmol dm^−3^; and *c_Ligand_* = 2.0 mmol dm^−3^.

Analyzing the Fe^3+^/*TXA*^−^ system reported in Figure 6, a net prevalence of the metal/chloride complexes that reach ~ 79% of formation can be noticed; the formation of the complexes of the other inorganic anions is limited and does not exceed for CO_3_^2−^ and PO_4_^3−^ 5%. The complexes formed by *TXA*^−^ with Fe^3+^ reach 10%.

## 3. Materials and Methods

### 3.1. Chemicals

The Fe^3+^ solutions were prepared by weighing the Fe(NO_3_)_3_∙6H_2_O and the FeCl_3_∙6H_2_O salts. The purity of the Fe^3+^ salts was checked by means of titrations with EDTA standard solutions [33,34]. The tranexamic acid (*TXA^−^*), indole-3-acetic acid (*IAA^−^*), and aminomethylphosphonic (*AMPA*^2−^) acid solutions were prepared without any further purification. Their purity, always > 99.5%, was verified using alkalimetric titrations. Hydrochloric acid and sodium hydroxide solutions, prepared from concentrated ampoules, were standardized using sodium carbonate and potassium biphtalate, respectively; the salts were previously dried in an oven at *T* = 383.15 K for 2 h. NaOH solutions were stored in dark bottles and preserved from CO_2_ using soda lime traps. The ionic medium aqueous solutions, namely NaNO_3_ and NaCl, were prepared by weighing the pure salt, pre-dried in an oven at *T* = 383.15 K. Ultrapure water (conductivity < 0.1 *μ*S cm^−1^) and grade A glassware were used for the preparation of each solution. For more details, see Table 3.

### 3.2. Potentiometric Equipment and Procedure

Potentiometric titrations were performed using an automatic titration system consisting of a Metrohm model 809 titrando coupled with a Metrohm 800 Dosino dispenser connected to a PC that controlled titrant delivery, data acquisition, and electromotive (*e.m.f.*) stability by means of the Metrohm TIAMO 2.5 software (Metrohm AG, Herisau, Switzerland). The system was equipped with a Metrohm 750 combined glass electrode (Metrohm AG, Herisau, Switzerland). The estimated accuracy of the potentiometric system is ±0.15 mV for *e.m.f.* and ±0.002 cm^3^ for titrant volume readings. The experiments were performed by titrating with standard NaOH, 25 cm^3^ of the solution containing a known amount of ligand, Fe^3+^, HCl to regulate the pH, and an ionic medium solution (NaNO_3_ or NaCl) in order to obtain the desired ionic strength values. More experimental details are reported in Table S1. All the measurements were performed in thermostated cells at *T* = 298.15 ± 0.15 K under magnetic stirring. Presaturated N_2_ was bubbled into the solution to remove O_2_ and CO_2_ inside. For each measurement, independent titrations of HCl with standard NaOH were performed to determine the standard electrode potential (E^0^) and the ionic product of water (*pK_w_*) at the same experimental conditions regarding the temperature and ionic strength of the experiments.

In the case of the competitive measurements with EDTA, the same experimental conditions of the simple Fe^3+^/*AMPA*^2−^ system were used, except for the simultaneous presence of aminopolycarboxylic acid in different amounts.

### 3.3. Spectrophotometric Apparatus and Procedure

A UV–Vis spectrophotometer (Varian, Cary 50 model; Agilent Technologies, Santa Clara, CA, USA), equipped with an optic fiber probe with a 1 cm path length, was used for carrying out the Fe^3+^/indole-3-acetic acid measurements in the wavelength range 200 ≤ λ/nm ≤ 500. The instrument was connected to a computer, and the absorbance (A) signal vs. wavelength (λ/nm) acquisition was performed employing the Varian Cary WinUV software. A combined glass electrode (Thermo-Orion, Ross type 8102; Thermo Fisher Scientific Inc., Waltham, MA, USA) connected to a potentiometer was also used for the potentiometric data recording. In total, 25 cm^3^ of the measurement solutions were titrated with NaOH 0.1035 mol dm^−3^ delivered by means of a Metrohm 665 automatic burette. In order to avoid the possible presence of atmospheric oxygen and carbon dioxide, before each experiment, presaturated N_2_ was bubbled in the solutions for at least 5 min. During the titrations, a magnetic stirrer provided to keep the homogeneity of the solutions. The Fe^3+^/*IAA*^−^ investigations were performed both in the absence of an ionic medium (*I* = 0.010 mol dm^−3^ in HCl) and at 0.15 ≤ *I*/mol dm^−3^ ≤ 1.00 in NaCl_(aq)_ and *T* = 298.15 K.

The measurement solutions contained the ligand (0.017 ≤ *c*_IAA_/mmol dm^−3^ ≤ 0.037) and the metal cation (0.017 ≤ *c*_Fe_/mmol dm^−3^ ≤ 0.02), as well as hydrochloric acid (*c*_HCl_ = 0.0103 mol dm^−3^) and, in some conditions, the ionic medium to reach the selected ionic strength condition. The pH range investigated was ~ 2.0–11.0, without the formation of precipitate in the measurement solutions. To be sure that no sparingly soluble species had formed, after each experiment, these solutions were centrifuged at 15,000 rot m^−1^ and *T* = 298.15 K for 15 min, without observing solid-to-supernatant separation. In addition, the centrifuged solutions were also irradiated using a laser source, and no possible light scattering was observed.

### 3.4. Calculations

The BSTAC computer program [20] was employed to process the potentiometric data, allowing us to calculate all the parameters of the acid–base titrations, namely the standard electrode potential (E^0^), the junction potential (*J*_a_), the ionic product of water (*pK*_w_), the analytical concentration of each component, and the formation constants of the Fe^3+^/ligand (*L*^n−^) complex species. The UV-Vis data analysis was performed by means of the HypSpec 2008 program [21].

The equilibria concerning the formation of the Fe^3+^/*L*^n−^ (protonated or hydrolytic) species can be expressed by the means of the following general equations:

*p* Fe^3+^ + *q L*^n−^ + *r* H^+^ = Fe*_p_L_q_*H*_r_^(3p+r−nq)^                   β_pqr_* (2)

Fe^3+^ + *L^n−^* + *r* H_2_O = Fe*L*(OH)*_r_^(3−r−n)^* + *r* H^+^           *β_11-r_* (3)

Since the formation of the Fe^3+^/*L*^n−^ species was investigated at different ionic strengths, the dependence of the equilibrium constants on *I*/mol dm^−3^ was modeled by means of an extended Debye–Hückel type equation, Equation (4):

log*β* = log*β*^0^ − z*·*DH* + *C*·*I* (4)

where log*β*^0^ is the formation constant at infinite dilution, *DH* = 0.51 (*I*^1/2^/(1 + 1.5*I*^1/2^)) is the Debye–Hückel term, and *C* is an empirical parameter for the dependence of the formation constant on the ionic strength (*I*).

The numerical value of the *C* parameter is dependent on the stoichiometry of the reaction and on the charge of the components that are involved in the formation reaction, and can be expressed by the following equation:

(5) $$C\;=c_{0}\cdot p^{*}+c_{1}\cdot z^{*}$$

where p* = Σ (stoich. coeff.)_reactants_ − Σ (stoich. coeff.)_products_ and z* = Σ (charge)^2^_reactants_ − Σ (charge)^2^_products_.

The conversion of the ionic strength and equilibrium constants from the molar to the molal concentration scale [35] allows us to express Equation (4) as reported in Equation (6), which is a simplified expression of the SIT model [36,37,38], where Δ*ε* ≅ *C* parameter of Equation (4) and *I*_m_ is the ionic strength expressed in molal concentration scale:

log*β* = log*β*^0^ − z*·*DH* + Δ*ε*·*I_m_* (6)

The use of the simplified SIT approach reported by means of Equation (6) allows us to calculate the specific ion interaction coefficients of the Fe^3+^/ligand species.

The spectrophotometric data were processed by means of the HypSpec program [21], which calculates the stability constant and the molar absorbance of the single species, when the analytical concentrations of the reagents are known. The LIANA program [20] was employed to study the dependence on ionic strength of the formation constants of the complex species and for the calculation of their values at infinite dilution and at the desired ionic strengths. By using LIANA program, the *C* and Δ*ε* parameters of Equations (4) and (6) were also calculated for each Fe^3+^/*L*^n−^ complex. The HySS program [39] was employed to draw the distribution diagram of the species and to calculate their formation percentage at the different experimental conditions (i.e., pH and ionic strength). Since, during the investigations of the Fe^3+^/*TXA*^−^ and Fe^3+^/*AMPA*^2−^ systems, the formation of the Fe(OH)_3(s)_ was observed at different experimental conditions, its solubility product [19] was considered during the simulation to obtain the distribution diagrams.

## 4. Conclusions

The results obtained from the elaboration and modeling of the experimental data highlight that Fe^3+^ has a selective ability to interact with tranexamic (*TXA*^−^), indole-3-acetic (*IAA*^−^), and aminomethylphosphonic (*AMPA*^2−^) acids. In the case of *TXA*^−^ and *AMPA*^2−^, the pH range of investigation was limited by the formation of the Fe(OH)_3(s)_ sparingly soluble species at pH~5.

Concerning *IAA*^−^, to avoid the formation of the sparingly soluble species, the studies were performed by spectrophotometry at lower concentrations of components with respect to the potentiometric ones, allowing for investigations in the pH range 2–11.

Different speciation models were obtained, and the stability of the Fe^3+^/ligand complexes resulted to be quite different, in dependence on the charges involved in the formation of the species. A comparison of the stability constants of the common Fe*L* species allowed us to obtain a fairly linear variation with respect to the z* parameter, with a correlation coefficient of 0.999. As an example, at *I* = 1.00 mol dm^−3^ and *T* = 298.15 K, for the ML species (M = Fe^3+^ and *L* = *IAA*^−^ or *AMPA*^2−^), log*K*_FeIAA_= 6.55 and log*K*_FeAMPA_= 14.84, highlighting that the phosphonate ligand is the best chelator towards Fe^3+^.

The modeling of the stability constants determined at different ionic strengths was performed by means of the Debye–Hückel type equation (Equation(4)) and the SIT approach (Equation(6)), allowing for the calculation of the *C* and Δ*ε* parameters, the thermodynamic stability constants (i.e., at infinite dilution), and the corresponding values at desired ionic strengths.

The effective sequestering ability of each ligand towards Fe^3+^, quantified at different experimental conditions (i.e., pH and ionic strength) by means of the pL_0.5_ parameter, allowed us to observe some important evidence, namely, significant change with experimental conditions (ionic strength and pH) variations. This confirmed that pL_0.5_ is a very important tool when comparing metal–ligand systems with different speciation models.

Considering the three systems and comparing their sequestering ability towards Fe^3+^ (Table S2 and Figure S5), *AMPA*^2−^ is the best chelator towards Fe^3+^, with pL_0.5_ values of 2.15, 4.00, and 4.89, at *I* = 1.0 mol dm^−3^ and pH = 5, for *IAA*^−^, *TXA*^−^, and *AMPA*^2−^, respectively.

To complete this study, some simulations on the distribution of Fe^3+^/*TXA*^−^; Fe^3+^/*AMPA*^2−^, and Fe^3+^/*IAA*^−^ species in multicomponent solutions (rainwater, seawater, urine, saliva and plasma) were performed, where the simultaneous presence of many other components and secondary equilibria complicated the investigation.

This allowed us to gain a more realistic representation of real system conditions, where the natural components (often at concentrations several orders of magnitude greater than the metal and ligands considered) interact not only with each other (internal equilibria), but also with the components to be studied (Fe^3+^, *TXA*^−^, *IAA*^−^, and *AMPA*^2−^, in our case). For example, in the case of the model used to simulate urine, composed of six cations and seven anions, 110 different equilibria must be considered by adding the Fe^3+^ hydrolysis constants, the protonation of the ligands; in the case of the Fe^3+^/*TXA*^−^ system, 123 different species/equilibria and formation constants were considered in the input; and in the case of blood plasma, 64 different equilibria.

The information obtained from these simulations, as represented by the pie charts, highlights how much the studies performed in the laboratory and performed in an electrolytic solution containing a single background salt can deviate from real conditions. In each case (single-electrolyte solution and multicomponent solution) the same sequestering trend was observed, namely *AMPA*^2−^ > *TXA*^−^ > *IAA*^−^.

The results obtained here are of fundamental importance both for biological and environmental problems due to the overloading of Fe^3+^, and also because knowledge of the thermodynamic parameters of the interactions and the distribution of the different metal–ligand species at the different experimental conditions can be useful to optimize the transport or remediation procedures of these components.

## Figures and Tables

**Table 1:** Experimental formation constants (log*β* ^(a)^) of Fe^3+^/*L* species in Na^+^ media at different ionic strengths and *T* = 298.15 K.

	*Ī* ^(b)^/mol dm^−3^			
Species	0.150	0.481	0.720	0.953
	NaNO_3(aq)_			
Fe(*TXA*)H^3+^	14.84 ± 0.01 ^(c)^	15.00 ± 0.01	15.36 ± 0.01	15.90 ± 0.04
Fe(*TXA*)_2_^+^	22.15 ± 0.03	22.46 ± 0.03	23.07 ± 0.02	23.18 ± 0.05
Fe(*TXA*)(OH)^+^	8.99 ± 0.02	8.82 ± 0.03	9.03 ± 0.04	9.88 ± 0.04
Fe(*TXA*)(OH)_2(aq)_	5.05 ± 0.02	5.04 ± 0.02	5.28 ± 0.02	6.30 ± 0.04
	0.010	0.144	0.501	0.998
	NaCl_(aq)_			
Fe(*IAA*)^2+^	6.52 ± 0.01 ^(c)^	6.47 ± 0.04	6.30 ± 0.01	6.54 ± 0.06
		0.105	0.486	0.949
	NaCl_(aq)_			
Fe(*AMPA*)H_2_^3+^		24.30 ± 0.04 ^(c)^	24.59 ± 0.04	22.79 ± 0.05
Fe(*AMPA*)H^2+^		21.08 ± 0.04	21.36 ± 0.01	19.30 ± 0.05
Fe(*AMPA*)^+^		15.07 ± 0.03	15.88 ± 0.04	13.49 ± 0.06

^(a)^ log*β* values refer to the equilibria of Equations. (2) and (3). ^(b)^ Average ionic strength. ^(c)^ ±Std. Dev.

**Table 2:** Thermodynamic formation constants of the Fe^3+^/ligand species and parameters for the dependence on ionic strength in NaCl_(aq)_ and *T* = 298.15 K.

Species	C ^(a)^	Δε ^(a)^	log*β*^0 (b)^	log*β* ^(b)^			
			*I/*mol dm^−3^				
			0	0.15	0.50	0.75	1.00
Fe(*TXA*)H^3+ (c)^	0.80 ± 0.04 ^(d)^	1.15 ± 0.13 ^(d)^	14.94 ± 0.02 ^(d)^	14.81	14.99	15.15	15.33
Fe(*TXA*)_2_^+^	2.24 ± 0.08	2.44 ± 0.11	22.98 ± 0.05	22.07	22.35	22.74	23.18
Fe(*TXA*)(OH)^+^	1.24 ± 0.08	1.59 ± 0.18	9.64 ± 0.04	8.82	8.86	9.03	9.24
Fe(*TXA*)(OH)_2_^0^ _(aq)_	2.21 ± 0.07	2.29 ± 0.17	5.43 ± 0.04	4.76	5.13	5.55	6.01
Fe(*IAA*)^2+^	0.84 ± 0.07	0.82 ± 0.07	6.92 ± 0.04	6.30	6.30	6.41	6.55
Fe(*AMPA*)H_2_^3+^	−0.336 ± 0.006	−0.36 ± 0.01	26.17 ± 0.02	25.37	24.95	24.76	24.61
Fe(*AMPA*)H^2+^	−0.17 ± 0.02	−0.18 ± 0.04	20.35 ± 0.05	19.08	18.52	18.30	18.14
Fe(*AMPA*)^+^	−0.022 ± 0.007	−0.026 ± 0.008	17.31 ± 0.05	15.81	15.20	14.99	14.84

^(a)^ Parameters for the dependence of log*β* on ionic strength determined by means of Equations (4) and (6). ^(b)^ Formation constants (log*β*^0^ and log*β*) refer to the equilibria in Equations (2) and (3) in molar scale; ^(c)^ in NaNO_3(aq)_; ^(d)^ ±Std. Dev.

**Table 3:** Chemicals used in this work, purchased from Merck (Darmstadt, Germany). Purity (mass) as stated by the supplier.

Chemical	CAS n°	Purification	Assay (% wt.)
Hydrochloric acid	7647-01-0	No	37%
Sodium hydroxide	1310-73-2	No	≥97%
Sodium carbonate	497-19-8	No	≥99.5%
Potassium phthalate monobasic	877-24-7	No	≥99.5%
Sodium nitrate	7631-99-4	No	≥99.0%
Sodium chloride	7647-14-5	No	≥99%
Iron(III) nitrate nonahydrate	7782-61-8	No	≥98%
Iron(III) chloride hexahydrate	10025-77-1	No	≥98%
Tranexamic acid	1197-18-8	No	>99.5%
Indole-3-acetic acid	87-51-4	No	>99.5%
Aminomethylphosphonic acid	1066-51-9	No	>99.5%

## Data Availability

Data will be made available on request.

## Supplementary Materials

The following supporting information can be downloaded at: https://www.mdpi.com/article/10.3390/molecules30142991/s1, Table S1. Experimental conditions of the systems studied at *T* = 298.15 K; Table S2. pL_0.5_ values calculated using Equation (1) for the different Fe^3+^/ligand systems at various pHs, ionic strengths and supporting electrolytes; Table S3. Stability constants of the main natural components of the rainwater and Fe^3+^/*AMPA*^2−^ species at *I* = 0.010 mol dm^−3 a)^ and *T* = 298.15 K; Table S4. Stability constants of the components of the urine and Fe^3+^/ligands species; Table S5. Stability constants of the components of the saliva and Fe^3+^/ligands species; Table S6. Stability constants of the components of the plasma and Fe^3+^/*TXA*^−^ species; Figure S1. Distribution diagram of the Fe^3+^/*TXA*^−^ species in NaNO_3(aq)_, at *I* = 0.15 mol dm^−3^ and *T* = 298.15 K. (Charges omitted for simplicity). Experimental conditions: *c*_Fe_ = 2.15 mmol dm^−3^, *c_TXA_* = 5.87 mmol dm^−3^ and *c*_Cl_ = 5.58 mmol dm^−3^; Figure S2. UV-Vis spectrophotometric titration curves of Fe^3+^/3-indoleacetic acid species, *c*_Fe_ = 0.02 mmol dm^−3^ and *c_IAA_* = 0.02 mmol dm^−3^, at *T* = 298.15 K and different pH values, (a) in absence of ionic medium (*I* = 0.01 mol dm^−3^ by HC_l(aq)_) and (b) in NaCl_(aq)_ at *I* = 1.00 mol dm^−3^; Figure S3. Distribution diagram of Fe^3+^/*IAA*^−^ at *I* = 0.15 mol dm^−3^ in NaCl_(aq)_. (Charges omitted for simplicity). Experimental conditions: *c*_Fe_ = 0.02 mmol dm^−3^; *c_IAA_* = 0.02 mmol dm^−3^ and *c*_Cl_ = 16.7 mmol dm^−3^; Figure S4. Distribution diagram of the Fe^3+^/*AMPA*^2−^ species in NaCl_(aq)_, at *I* = 0.10 mol dm^−3^ and *T* = 298.15 K. (Charges omitted for simplicity). Experimental conditions: *c*_Fe_ = 1.52 mmol dm^−3^, *c_AMPA_* = 3.02 mmol dm^−3^ and *c*_Cl_ = 110.1 mmol dm^−3^; Figure S5. Sequestration diagram of *TXA*^−^, *IAA*^−^ and *AMPA*^2−^ towards Fe^3+^ in Na^+^ media, at *I* = 1.00 mol dm^−3^, pH = 5.0 and *T* = 298.15 K. pL_0.5_: 4.89 (*AMPA*^2−^); 4.00 (*TXA*^−^); 2.15 (*IAA*^−^).
