# Supplementary material for: Ligands of Biological and Environmental Interest as Sequestering Agents for Fe3+ in Aqueous Solution: A Speciation Study of Natural Fluids
Source: Molecules. 2025 Jul 16;30(14):2991. doi: 10.3390/molecules30142991 (PMC12299677; doi:10.3390/molecules30142991)
Supplement: Supplementary file 1 [file molecules-30-02991-s001.zip › molecules-3739253-supplementary.pdf]

Supporting Information

Ligands of biological and environmental interest as sequestering agents for Fe^{3+} in aqueous solution: A speciation study for natural fluids

Anna Irto[†], Ileana Ielo[†], Clemente Bretti, Francesco Crea[*], Concetta De Stefano, Rosalia Maria Cigala

Dipartimento di Scienze Chimiche, Biologiche, Farmaceutiche ed Ambientali, Università di Messina, Viale F. Stagno d'Alcontres, 31 – 98166 Messina, Italy

Table S1. Experimental conditions of the studied systems at T = 298.15 K

Systems	c_{Fe} [a)]	c_{Ligand} [a)]	pH range	I [b)]	Ionic medium	Techniques
Fe^{3+}/TXA^-	1.4-2.1	1.4-7.1	2.2-5.3	0.15-1.00	$NaNO_3$	Pot
Fe^{3+}/IAA^-	0.02	0.02-0.04	2.0-11.0	0.15-1.00	NaCl	UV-Vis
$Fe^{3+}/AMPA^{2-}$	1.1-2.1	1.1-6.5	1.9-5.3	0.10-1.00	NaCl	Pot

[a)] in mmol dm^{-3}; [b)] in mol dm^{-3}.

[*]Corresponding author: Francesco Crea, email: fcrea@unime.it.

[†]The two authors contributed equally to the manuscript.

Table S2. $pL_{0.5}$ values calculated using eq. (1) for the different Fe^{3+}/ligand systems at various pHs, ionic strengths and supporting electrolytes

Ligand	*I*/mol dm^{-3}	pH	$pL_{0.5}$	Ligand	*I*/mol dm^{-3}	pH	$pL_{0.5}$
TXA$^-$	0.15	2.0	1.90	*IAA*$^-$	0.15	4.0	3.59
	0.15	3.0	2.50		0.15	5.0	1.97
	0.15	4.0	2.72		0.15	6.0	< 0.50
	0.15	5.0	4.90		0.15	7.4	< 0.50
	0.50	2.0	2.01		0.50	5.0	1.9
	0.50	3.0	2.43		0.50	7.4	< 0.50
	0.50	4.0	3.63		1.00	5.0	2.15
	0.50	5.0	4.65		1.00	7.4	< 0.50
	0.75	2.0	2.25	*AMPA*$^{2-}$	0.10	2.0	8.95
	0.75	3.0	2.47		0.10	3.0	8.39
	0.75	4.0	3.37		0.10	4.0	7.28
	0.75	5.0	4.58		0.10	5.0	6.15
	1.00	2.0	1.76		0.50	2.0	9.57
	1.00	3.0	1.86		0.50	3.0	9.19
	1.00	4.0	2.79		0.50	4.0	8.08
	1.00	5.0	4.00		0.50	5.0	7.00
IAA$^-$	0.01	5.0	1.23		1.00	2.0	7.80
	0.01	7.4	< 0.50		1.00	3.0	7.37
	0.15	2.0	4.16		1.00	4.0	6.07
	0.15	3.0	4.48		1.00	5.0	4.89

Table S3. Stability constants of the main natural components of the rainwater and Fe^{3+}/$AMPA^{2-}$ species at I = 0.010 mol dm^{-3} [a] and T = 298.15 K.

Species	logβ	Species	logβ
NaOH	-13.3[b]	Fe(OH)	-2.36[c]
NaCl	-0.5	$Fe(OH)_2$	-5.09
$Na(SO_4)$	0.46	$Fe_2(OH)_2$	-3.48
KCl	-0.48	$Fe_3(OH)_4$	-4.17
$K(SO_4)$	0.55	$Fe_{12}(OH)_{34}$	-50.82
MgCl	0.18		
$Mg(SO_4)$	1.65	FeCl	1.08[c, d]
CaCl	0.03	$FeCl_2$	1.6
$Ca(SO_4)$	1.6	FeCl(OH)	-0.51
$H(SO_4)$	1.79		
Mg(OH)	-11.5	$Fe(SO_4)$	3.3[e]
Ca(OH)	-12.5		
H_2O	-13.78	$Fe(CO_3)$	13.0[e]
KOH	-13.4		
$H(CO_3)$	9.85	Fe(*AMPA*)H_2	25.90[f]
$H_2(CO_3)$	15.97	Fe(*AMPA*)H	19.90
$Ca(CO_3)$	10.86	Fe(*AMPA*)	16.78
$Mg(CO_3)$	10.56		
$Na(CO_3)$	9.87		
$K(CO_3)$	9.79		

[a] Estimated mean ionic strength value of rainwater; [b] Recalculated values from [1]; [c] Recalculated values from [2]; [d] Recalculated values from [3]; [e] Recalculated values from [4]; [f] Recalculated values from this work.

Table S4. Stability constants of the components of the urine and Fe^{3+}/ligands species

Species	logβ	Species	logβ	Species	logβ
H_2O	-13.83[a)]	H*IAA*	Unpublished data	H_2*TXA*	Unpublished data
NaOH	-13.59	Ca^{2+}/*IAA*$^-$	Unpublished data	Ca*TXA*	Unpublished data
KOH	-13.65	Mg^{2+}/*IAA*$^-$	Unpublished data	Mg*TXA*	Unpublished data
CaOH	-12.85	Fe(*IAA*)	6.33 [e)]	Fe(*TXA*)H	14.84 [e)]
Mg(OH)	-11.72	HOx	3.9[b)]	Fe(*TXA*)$_2$	22.15
$(NH_4)(OH)$	-9.25	H_2Ox	5.04	Fe(*TXA*)(OH)	8.99
HCit	5.87	NaOx	0.47	Fe(*TXA*)$(OH)_2$	5.05
H_2Cit	10.26	NaOxH	3.59	$H(PO_4)$	11.88[a)]
H_3Cit	13.21	KOx	0.26	$H_2(PO_4)$	18.76
NaCit	1.0	KOxH	3.39	$H_3(PO_4)$	20.75
Na_2Cit	1.42	MgOx	2.73	$Na(PO_4)$	0.95
NaCitH	6.37	CaOx	2.38	$Na(PO_4)H$	12.6
NaCitH_2	10.29	FeOx	8.0[b)]	$Na(PO_4)H_2$	18.87
Na_2CitH	5.97	FeOxH	7.8	$Na_2(PO_4)$	1.77
KCit	0.87	FeOx(OH)	2.0	$Na_2(PO_4)H$	12.32
K_2Cit	0.97	Fe$(Ox)_2$	14.2	$K(PO_4)$	0.85
KCitH	6.17	Fe$(Ox)_3$	19.0	$K(PO_4)H$	12.39
KCitH_2	10.19	NaCl	-0.43	$K(PO_4)H_2$	18.75
K_2CitH	5.97	KCl	-0.44	$K_2(PO_4)$	1.41
NaKCit	1.52	MgCl	0.18	$K_2(PO_4)H$	12.33
NaKCitH	6.34	CaCl	0.03	$Ca(PO_4)H$	13.79
MgCit	3.59	$(NH_4)Cl$	-0.46	$Ca(PO_4)H_2$	19.77
MgCitH	7.54	$(SO_4)H$	1.67	$Mg(PO_4)H$	13.93
MgCitH_2	10.88	$Na(SO_4)$	0.48	$Mg(PO_4)H_2$	19.9
CaCit	3.79	$K(SO_4)$	0.55	$(NH_4)(PO_4)$	0.95
CaCitH	7.93	$Mg(SO_4)$	1.67	$(NH_4)(PO_4)H$	12.6
CaCitH_2	10.98	$Ca(SO_4)$	1.61	$(NH_4)(PO_4)H_2$	18.87
(NH_4)Cit	0.96	$(NH_4)(SO_4)$	0.94	$(NH_4)_2(PO_4)$	1.77
(NH_4)CitH	6.35	$Fe(SO_4)$	2.9[b)]	$(NH_4)_2(PO_4)H$	12.3
$(NH_4)_2$Cit	1.01	$Fe(SO_4)_2$	3.83	$NaK(PO_4)$	1.95
(NH_4)CitH_2	10.32	H(UA)	5.26[b)]	$Na(NH_4)(PO_4)$	2.12
$(NH_4)_2$CitH	5.82	HU	0.16[b)]	$K(NH_4)(PO_4)$	1.95
$Na(NH_4)$Cit	1.52	HU_2	-0.55	$NaK(PO_4)H$	12.44
$Na(NH_4)$CitH	7.41	UCitH	4.88[b)]	$Na(NH_4)(PO_4)H$	12.42
$K(NH_4)$Cit	1.52	UCitH_2	9.89	$K(NH_4)(PO_4)H$	12.44
$K(NH_4)$CitH	7.41	UCitH_3	12.91	$Fe(PO_4)$	10.24
FeCit	11.5[b)]	CaU	-0.81[b)]	$Fe(PO_4)H$	20.5
FeCitH	12.1	MgU	-0.31[b)]	$Fe(PO_4)H_2$	22.07
Fe(OH)Cit	9.0	$U(SO_4)H$	0.97[b)]	H(*AMPA*)	9.86[d)]
FeCit$_2$	16.5	$U(PO_4)H_2$	17.73	H_2(*AMPA*)	15.22
Fe(OH)	-2.66[c)]	$U(PO_4)H_3$	20.39	Ca(*AMPA*)H_2	17.12[d)]
$Fe(OH)_2$	-5.45	FeU	0.78[b)]	Ca(*AMPA*)H	11.33
$Fe_2(OH)_2$	-3.47	FeU_2	1.04	Mg(*AMPA*)H_2	17.25[d)]
$Fe_3(OH)_4$	-4.43	FeU_3	0.994	Mg(*AMPA*)H	12.35
$Fe_{12}(OH)_{34}$	-55.59	H*TXA*	Unpublished data	Fe(*AMPA*)H_2	25.48[e)]
				Fe(*AMPA*)H	19.24
				Fe(*AMPA*)	15.99

Abbreviations: Cit = Citric acid, Ox = Oxalic acid; UA = Uric acid; U = Urea.

[a)] Recalculated values from [1]; [b)] Recalculated values from [4]; [c)] Recalculated values from [2]; [d)] Recalculated values from [5]; [e)] Recalculated values from this work.

Table S5. Stability constants of the components of the saliva and Fe^{3+}/ligands species

Species	logβ	Species	logβ	Species	logβ
H_2O	-13.83[a)]	$Fe_3(OH)_4$	-4.43	Ca*TXA*	Unpublished data
NaOH	-13.59	$Fe_{12}(OH)_{34}$	-55.59	Mg*TXA*	Unpublished data
KOH	-13.65	H*IAA*	Unpublished data	Fe(*TXA*)H	14.84[e)]
CaOH	-12.85	Ca^{2+}/IAA^{-}	Unpublished data	$Fe(\mathit{TXA})_2$	22.15
Mg(OH)	-11.72	Mg^{2+}/IAA^{-}	Unpublished data	Fe(*TXA*)(OH)	8.99
$(NH_4)(OH)$	-9.25	$H(CO_3)$[a)]	9.98	$Fe(\mathit{TXA})(OH)_2$	5.05
HSCN	-1.65	$H_2(CO_3)$	16.17	Fe(*IAA*)	6.33[e)]
NaSCN	-0.51	$Na(CO_3)$	0.82	$H(PO_4)$[a)]	11.88[a)]
KSCN	-0.49	$Na(CO_3)H$	10.0	$H_2(PO_4)$	18.76
MgSCN	-1.17	$K(CO_3)$	0.61	$H_3(PO_4)$	20.75
CaSCN	-1.17	$K(CO_3)H$	9.9	$Na(PO_4)$	0.95
$(NH_4)SCN$	-0.51	$Ca(CO_3)$	2.52	$Na(PO_4)H$	12.6
FeSCN	2.4	$Ca(CO_3)H$	10.89	$Na(PO_4)H_2$	18.87
$Fe(SCN)_2$	3.6	$Mg(CO_3)$	2.21	$Na_2(PO_4)$	1.77
HF	2.97	$Mg(CO_3)H$	10.68	$Na_2(PO_4)H$	12.32
HF_2	3.53	$(NH_4)(CO_3)$	0.82	$K(PO_4)$	0.85
NaF	-0.57	$(NH_4)(CO_3)H$	10.0	$K(PO_4)H$	12.39
KF	-0.55	$Fe(CO_3)$	6.0	$K(PO_4)H_2$	18.75
MgF	1.34	$Fe(CO_3)H$	13.0	$K_2(PO_4)$	1.41
CaF	0.73	$Fe(CO_3)_2$	15.0	$K_2(PO_4)H$	12.33
$(NH_4)F$	-0.57	NaCl	-0.43	$Ca(PO_4)H$	13.79
FeF	5.45	KCl	-0.44	$Ca(PO_4)H_2$	19.77
FeF_2	9.64	MgCl	0.18	$Mg(PO_4)H$	13.93
FeF_3	12.4	CaCl	0.03	$Mg(PO_4)H_2$	19.9
HGly	9.69	$(NH_4)Cl$	-0.46	$(NH_4)(PO_4)$	0.95
H_2Gly	11.78	$(SO_4)H$	1.67	$(NH_4)(PO_4)H$	12.6
NaGly	-0.54	$Na(SO_4)$	0.48	$(NH_4)(PO_4)H_2$	18.87
NaGlyH	9.19	$K(SO_4)$	0.55	$(NH_4)_2(PO_4)$	1.77
KGly	-0.54	$Mg(SO_4)$	1.67	$(NH_4)_2(PO_4)H$	12.3
KGlyH	9.19	$Ca(SO_4)$	1.61	$NaK(PO_4)$	1.95
$(NH_4)Gly$	-0.54	$(NH_4)(SO_4)$	0.94	$Na(NH_4)(PO_4)$	2.12
$(NH_4)GlyH$	9.19	$Fe(SO_4)$	2.9[b)]	$K(NH_4)(PO_4)$	1.95
MgGly	1.06	$Fe(SO_4)_2$	3.83	$NaK(PO_4)H$	12.44
MgGlyH	9.71	HU	0.16[b)]	$Na(NH_4)(PO_4)H$	12.42
CaGly	1.16	HU_2	-0.55	$K(NH_4)(PO_4)H$	12.44
CaGlyH	9.88	CaU	-0.81[b)]	$Fe(PO_4)$	10.24[b)]
$Gly(SO_4)H$	10.75	MgU	-0.31[b)]	$Fe(PO_4)H$	20.5
GlyClH	11.61	$U(SO_4)H$	0.97[b)]	$Fe(PO_4)H_2$	22.07
GlyFH	11.8	$U(PO_4)H_2$	17.73	H(*AMPA*)	9.86[d)]
GlySCNH	11.61	$U(PO_4)H_3$	20.39	$H_2(\mathit{AMPA})$	15.22
FeGly	10.83	FeU	0.78[b)]	$Ca(\mathit{AMPA})H_2$	17.12[d)]
$FeGly_2$	20.48	FeU_2	1.04	Ca(*AMPA*)H	11.33
Fe(OH)	-2.66[c)]	FeU_3	0.994	$Mg(\mathit{AMPA})H_2$	17.25[d)]
$Fe(OH)_2$	-5.45	H*TXA*	Unpublished data	Mg(*AMPA*)H	12.35
$Fe_2(OH)_2$	-3.47	$H_2\mathit{TXA}$	Unpublished data	$Fe(\mathit{AMPA})H_2$	25.48[e)]
				Fe(*AMPA*)H	19.24
				Fe(*AMPA*)	15.99

Abbreviation: U = Urea.

[a)] Recalculated values from [1]; [b)] Recalculated values from [4]; [c)] Recalculated values from [2]; [d)] Recalculated values from [5]; [e)] Recalculated values from this work.

Table S6. Stability constants of the components of the plasma and Fe^{3+}/*TXA*$^{-}$ species

Species	logβ	Species	logβ	Species	logβ
H_2O	-13.83[a)]	$H(CO_3)$	9.98[a)]	$H(PO_4)$[a)]	11.88[a)]
NaOH	-13.59	$H_2(CO_3)$	16.17	$H_2(PO_4)$	18.76
KOH	-13.65	$Na(CO_3)$	0.82	$H_3(PO_4)$	20.75
CaOH	-12.85	$Na(CO_3)H$	10	$Na(PO_4)$	0.95
Mg(OH)	-11.72	$K(CO_3)$	0.61	$Na(PO_4)H$	12.6
NaCl	-0.43	$K(CO_3)H$	9.9	$Na(PO_4)H_2$	18.87
KCl	-0.44	$Ca(CO_3)$	2.52	$Na_2(PO_4)$	1.77
MgCl	0.18	$Ca(CO_3)H$	10.89	$Na_2(PO_4)H$	12.32
CaCl	0.03	$Mg(CO_3)$	2.21	$K(PO_4)$	0.85
Fe(OH)	-2.66[b)]	$Mg(CO_3)H$	10.68	$K(PO_4)H$	12.39
$Fe(OH)_2$	-5.45	$Fe(CO_3)$	6.0[c)]	$K(PO_4)H_2$	18.75
$Fe_2(OH)_2$	-3.47	$Fe(CO_3)H$	13.0	$K_2(PO_4)$	1.41
$Fe_3(OH)_4$	-4.43	$Fe(CO_3)_2$	15.0	$K_2(PO_4)H$	12.33
$Fe_{12}(OH)_{34}$	-55.59	H(THAM)	7.77[c)]	$Ca(PO_4)H_2$	19.77
$H(SO_4)$	1.67[a)]	$Fe(SO_4)$THAMH	2.5[c)]	$Mg(PO_4)H$	13.93
$Na(SO_4)$	0.48	H*IAA*	Unpublished data	$Mg(PO_4)H_2$	19.9
$K(SO_4)$	0.55	Ca^{2+}/*TXA*$^{-}$	Unpublished data	$NaK(PO_4)$	1.95
$Mg(SO_4)$	1.67	Mg^{2+}/*TXA*$^{-}$	Unpublished data	$NaK(PO_4)H$	12.44
$Ca(SO_4)$	1.61	Fe(*TXA*)H	14.84 [d)]	$Fe(PO_4)$	10.24[c)]
$Fe(SO_4)$	2.9[c)]	$Fe(TXA)_2$	22.15	$Fe(PO_4)H$	20.5
$Fe(SO_4)_2$	3.83	Fe(*TXA*)(OH)	8.99	$Fe(PO_4)H_2$	22.07
		$Fe(TXA)(OH)_2$	5.05		

Abbreviation: THAM = Tris(hydroxymethyl)aminomethane.

[a)] Recalculated values from [1]; [b)] Recalculated values from [2]; [c)] Recalculated values from [4]; [d)] Recalculated values from this work.

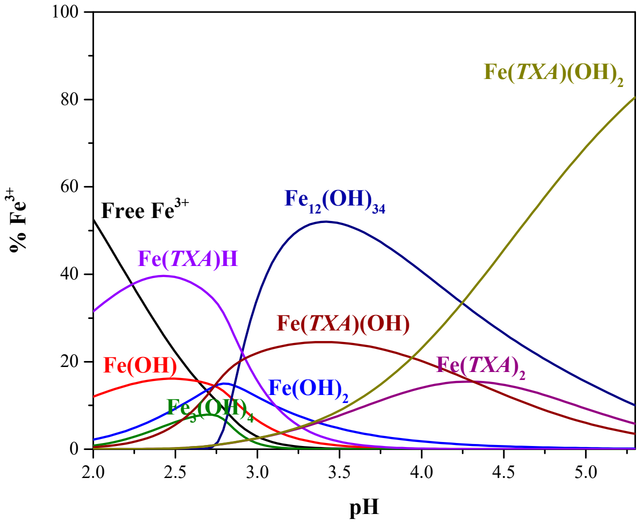

Figure S1. Distribution diagram of the Fe^{3+}/TXA^{-} species in $NaNO_{3(aq)}$, at $I = 0.15$ mol dm^{-3} and T = 298.15 K. (Charges omitted for simplicity)

Experimental conditions: c_{Fe} = 2.15 mmol dm^{-3}, c_{TXA} = 5.87 mmol dm^{-3} and c_{Cl} = 5.58 mmol dm^{-3}.

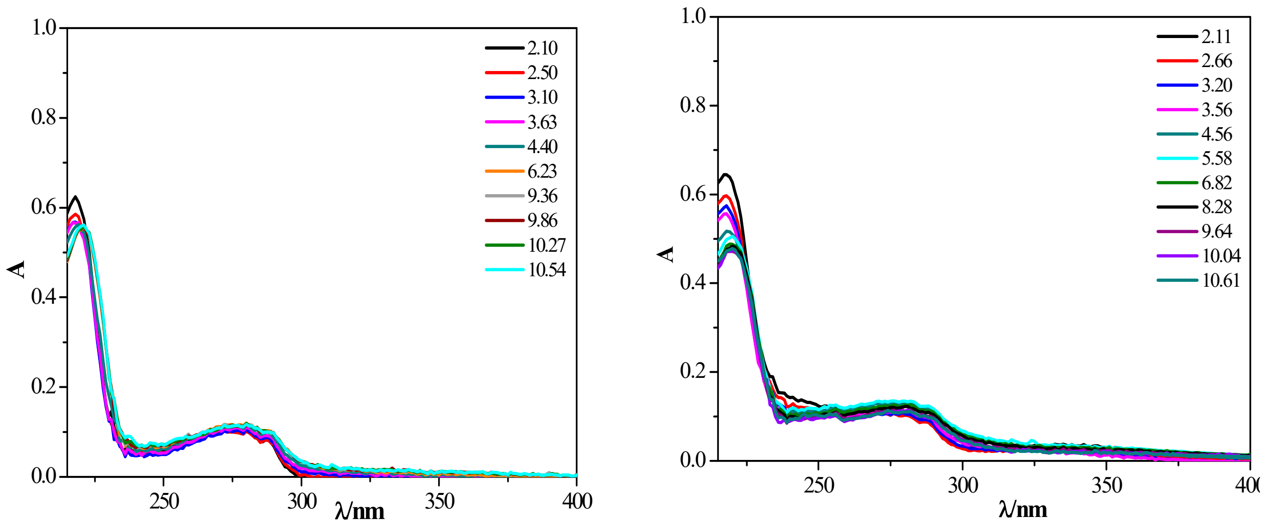

a) **b)**

Figure S2. UV-Vis spectrophotometric titration curves of Fe^{3+}/3-indoleacetic acid species, c_{Fe} = 0.02 mmol dm^{-3} and c_{IAA} = 0.02 mmol dm^{-3}, at T = 298.15 K and different pH values, **a)** in absence of ionic medium (I = 0.01 mol dm^{-3} by $HCl_{(aq)}$) and **b)** in $NaCl_{(aq)}$ at I = 1.00 mol dm^{-3}.

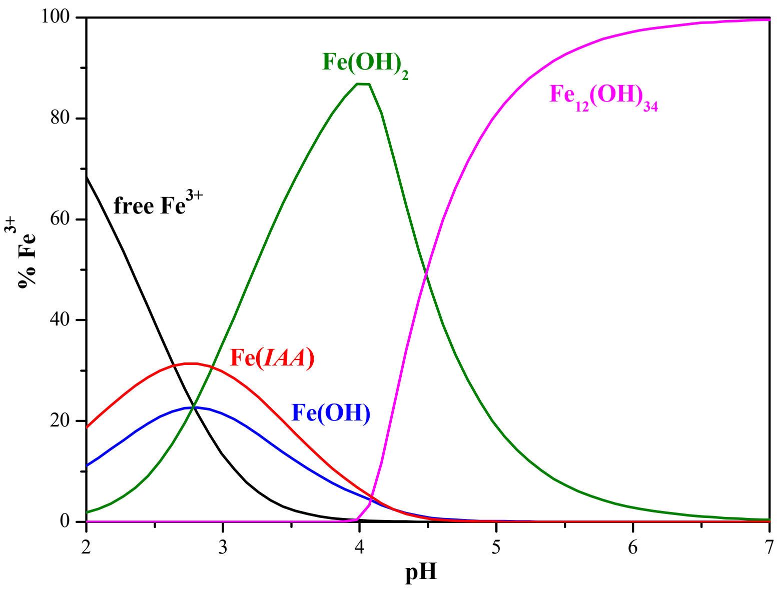

Figure S3. Distribution diagram of Fe^{3+}/*IAA*$^-$ at I = 0.15 mol dm^{-3} in $NaCl_{(aq)}$. (Charges omitted for simplicity)

Experimental conditions: c_{Fe} = 0.02 mmol dm^{-3}; c_{IAA} = 0.02 mmol dm^{-3} and c_{Cl} = 16.7 mmol dm^{-3}

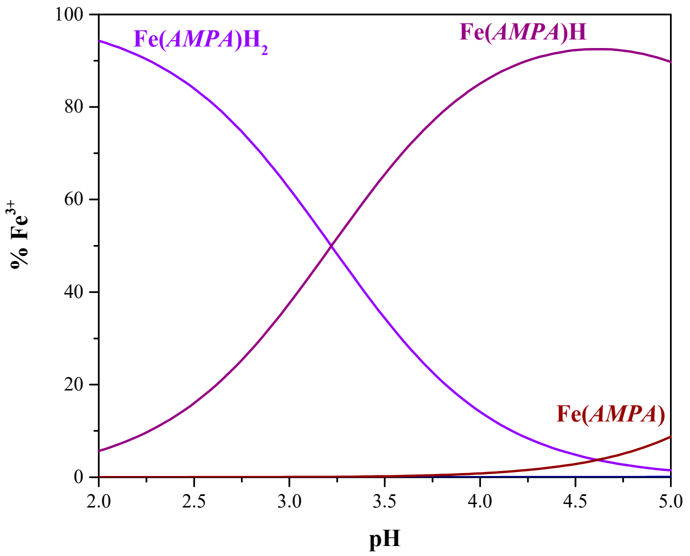

Figure S4. Distribution diagram of the Fe^{3+}/*AMPA*$^{2-}$ species in $NaCl_{(aq)}$, at I = 0.10mol dm^{-3} and T = 298.15 K. (Charges omitted for simplicity)

Experimental conditions: c_{Fe} = 1.52 mmol dm^{-3}, c_{AMPA} = 3.02 mmol dm^{-3} and c_{Cl} = 110.1 mmol dm^{-3}.

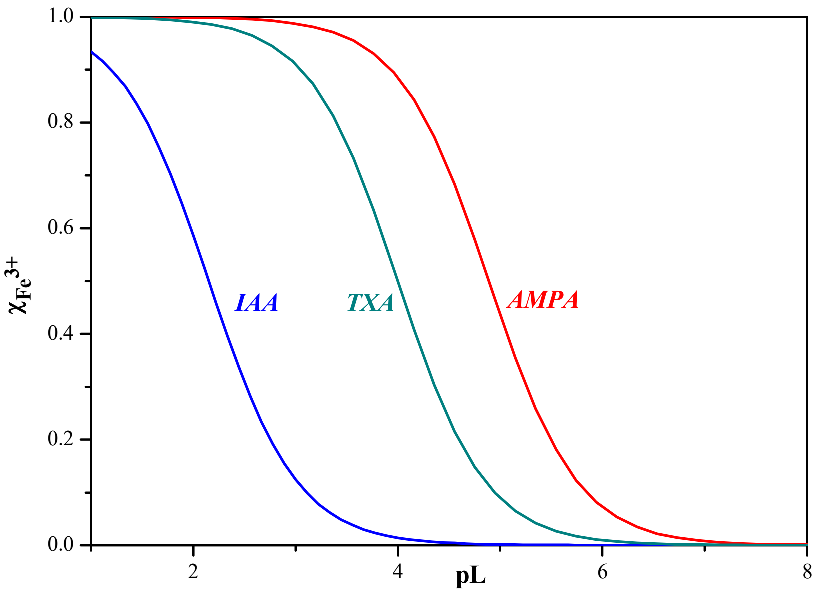

Figure S5. Sequestration diagram of *TXA*$^-$, *IAA*$^-$ and *AMPA*$^{2-}$ towards Fe^{3+} in Na^+ media, at I = 1.00 mol dm^{-3}, pH = 5.0 and T = 298.15 K. $pL_{0.5}$: 4.89 (*AMPA*$^{2-}$); 4.00 (*TXA*$^-$); 2.15 (*IAA*$^-$)

Bibliography for Supplementary Material

[1] F. Crea, C. De Stefano, D. Milea, A. Pettignano, S. Sammartano, Bioinorg. Chem. Appl. 2015 (2015) 12.
[2] A. Irto, R.M. Cigala, C. De Stefano, F. Crea, Journal of Molecular Liquids 391 (2023) 123361.
[3] P.L. Brown, C. Ekberg, Hydrolysis of Metal Ions, Wiley-VCH Verlag GmbH & Co. KGaA 2016.
[4] E.F. May, P.M. May, K. Murray, R. Darren, Joint Expert Speciation System; JESS Primer, 2019.
[5] R.M. Cigala, C. De Stefano, A. Irto, P. Lanzafame, G. Papanikolaou, F. Crea, Chemosphere 306 (2022) 135535.
